# Proceedings of the Memphis Medical Society

**Published:** 1899-03

**Authors:** 


### PROCEEDINGS OF THE MEMPHIS MEDICAL SOCIETY.

*Stated Meeting, January 17, 1899. Dr. B. F. Turner, President.*

Dr. W. B. Rogers read a paper on "Intra-scrotal Enlargements," especially with reference to their diagnosis. He adopted the following anatomical classification :

I. Enlargement of the cord alone, *i. e.*, from, or including, or surrounding the cord:

- a. Varicocele.
- b. Hydrocele of the cord—1. Encysted.
  2. Diffused.
- c. Hernia—1. Enterocele.
  2. Epiplocele.
- d. Hematocele.
- e. Spermatocele.

II. Enlargement of the testis alone.

- a. Inflammatory—1. Epididymitis.
  2. Orchitis.
- b. Non-inflammatory—1. Hydrocele. Simple.
  Congenital.
  Symptomatic.
  2. Hematocele.
- c. Sarcocele—Inflammatory (Simple).
  Syphilitic.
  Tubercular.
  Fatty.
  Cartilaginous.
  Malignant.

III. Enlargement of both cord and testis.

Hernia—1. Enterocele.
2. Epiplocele.

Under this head will come some of the other enlargements i fully developed. Diffused hydrocele of the cord is a collection of serum in the connective tissue. Encysted hydrocele of the cord occurs in the process of the tunica vaginalis.

For the purpose of the diagnosis we may divide these enlargements into the reducible and the irreducible. Of the enlargements of the cord, hernia and varicocele are reducible. When reduced, pressure will prevent the return of a hernia, but not a varicocele. Hydrocele and hematocele are irreducible: the former is transparent, the latter opaque. In negroes, where this test is of no value, a hypodermic may be used to make the diagnosis. Fatty tumor of the cord has occurred, but had best be let alone, unless large.

Of the enlargements of the testes alone, only one is reducible, viz., congenital hydrocele. Another division may be made of these enlargements into cystic and solid, the two being readily differentiated by puncture with a hypodermic. In uncomplicated tumors of the testis a diagnosis can generally be made without asking the patient a single question. The diagnostic features of inflammatory enlargements of the testis are sufficiently plain. Of the hydroceles, the simple form is due to a loss of balance in the blood-vessels of the parts, leading to an effusion of serum into the tunica vaginalis. Symptomatic hydrocele is due to a pathological condition in the testis. It is important to differentiate these two for therapeutic reasons.

Hematocele is due to traumatic ruptures of the large vessels around the epididymis. It is usually absorbed, but if not it may be laid open and laid out.

Of the solid enlargements of the testis, simple sarcocele can be diagnosed by the history and by exploration. It is a slow inflammatory process, with a deposit of fibrin in the connective tissue of the testicle. It forms a small, hard, painless tumor.

Syphilitic tumors do not get large, and are usually confined to the body of the testicle. Dr. Rogers cited a case in which the enlargement was double and confined to the epididymis, causing impotence. A cure was obtained by the iodides. Syphilitic tumors are painless and of slow growth.

Tuberculosis of the testis begins in the epididymis, usually the head, grows slowly and painlessly, and tends to become cheesy, the skin breaks and a fungous growth results.

Of fatty tumors one case is reported.

Cartilaginous tumors are found in the testis in common with the parotid and mammary glands, and in the testicle are generally associated with malignancy. This tumor is very hard.

Malignant tumors grow rapidly, and in the case of carcinoma cause the neighboring lymphatics to be enlarged. Carcinoma is painful, sarcoma is not. Carcinoma is said by a well-known author to be common in the young, but this is not Dr. Rogers's experience.

The features which belong to these growths in all localities and which separate them from each other, serve as diagnostic points for malignant tumors of testis.

Hernia involving cord and testis is generally not difficult to recognize.

Dr. Frank A. Jones has seen many cases of syphilitic sarcocele among the negroes at the East End Dispensary.

Dr. Wm. Krauss has had the opportunity of examining specimens of all sorts of tumors of the testicle, and a recent one submitted to him by Dr. Smythe was found to be a fibro-myoma, probably springing from the muscular tissue of the walls of the blood-vessel or of the vas deferens. Another case from the same operator proved to be purely fibrous, of inflammatory origin.

Dr. F. D. Smythe has seen an inflammatory enlargement of the cord quite often from sepsis following the operation for varicocele. In regard to the cases referred to by Dr. Krauss, the fibro-myoma was very large, extending up to the external ring, and had a colloid center. The fibrous enlargement was small and very hard, interfering with sexual intercourse and causing pain in the testis.

Dr. G. G. Buford mentioned a case where the patient rode home on a mule after having the testicle punctured with a hypodermic, and an orchitis was set up, for which he was held responsible. In another patient with a tubercular sarcocele, pain was quite a prominent symptom.

Dr. E. P. Sale called Dr. Rogers's attention to the omission of spermatocele, inserted above to make the table complete. This is a small, fluctuating tumor of the cord, containing "seminal seed."

Dr. R. B. Maury reported a case which was looked upon as "epididymitis or orchitis." It was of fifteen days' duration when he saw it, and containing fluid, was tapped, and the testicle found enlarged. There was no history of venereal disease, but of an effort on the part of the patient to catch himself and avoid a fall, and this was followed by pain in the testis. There was subsequently another slight injury, with reinflammation of the testicle and its final protrusion and destruction. Dr. Maury has seen ovarian pain follow just such an injury (indirect trauma).

Dr. Rogers referred briefly to the treatment of hydrocele. Injections of iodin, tannic acid, etc., cause restoration of the lost balance to the blood-vessels. The "open method" of free incision and packing causes obliteration of the sac; carbolic acid causes destruction and inflammation. In a case treated by iodin, carbolic acid, and the "open method," a recurrence was relieved by repeated injections. In reply to a question from Dr. Williams, he believed dermoid cysts are rarely met with in the testicle. In reply to a question from Dr. Sale, he had not used turpentine as an injection in hydrocele. In reply to a question from Dr. Moore, he does not think the injection of carbolic acid is attended by any danger of carbolic poisoning. After treatment by injection, some patients remain well four or five years and then the trouble recurs.

Dr. Smythe thinks carbolic acid cures by adhesion.

Dr. Sale has used turpentine and oil in one case by injection, bringing about a cure, but producing mild strangury. It did not cause much pain.

Dr. Krauss said that adhesions between the surfaces of the tunica vaginalis were not necessary for a cure, but only a closure of the openings in the serous membrane.

Dr. F. D. Smythe read a paper entitled, "A Plea against the Unwarranted Assault upon the Female Genitalia by Aspirants for Notoriety in the Practice of Obstetrics and Gynecology." Either through ignorance, desire to do something, or for a fee, practically normal pelvic and peritoneal structures are often attacked with disastrous results. Even when indications are present, operations undertaken by one not qualified to operate utterly fail to relieve the condition, if indeed they do not make it worse. He spoke specifically against the reckless curetting of puerperal uteri, needless vaginal examinations of virgins, useless removal of tubes and ovaries, improperly done plastic operations, and unnecessary gynecological "tinkering."

Dr. Edwin Williams is a believer in medical as opposed to surgical treatment in the majority of cases, and thinks the pelvic organs in virgins should always be examined by the rectum.

Dr. Alfred Moore spoke of the danger of breaking down, with a curette, the protecting leucocytic wall in the puerperal uterus and permitting septic infection. On the other hand, it is necessary, when the wall is infected, to remove it with the finger or dull curette. It is difficult to differentiate these cases. The uterine contents may be drawn off in a sterile tube, and submitted to bacteriological examination for the purpose of making a diagnosis.

Dr. Jones thinks Dr. Smythe is rather "ultra." Much of the trouble lies in the lack of preparation which alleged "specialists" experience.

Dr. Krauss approves of such iconoclasm when it emanates from one qualified to speak *ex cathedra*.

Dr. Buford agreed as to the evils of indiscriminate specialism. The danger of needless operations is based on improper diagnosis.

Dr. E. C. Ellett thinks Dr. Smythe's remarks might have a broader application than to any one specialty. Men frequently blossom as specialists after a six-weeks' course in a postgraduate school, and sometimes simply drop general practice and take up a specialty without any preparation or special study at all. Such men not only fail to become of any advantage to themselves or their unfortunate patients, but bring discredit upon the profession as a whole, and especially on the specialty which they presume to practice.

Dr. Sale believes in conservatism, but thinks Dr. Smythe is too radical in some things. In virgins it sometimes is absolutely necessary to make a vaginal examination. In obstetrics, too, the present "do nothing" plan is often inappropriate.

The President said that every man must at some time do his first operation, particularly in rural districts where it is not possible to always command skilled operators. In obstetrical work he favors frequent examinations under proper precautions.

Dr. Smythe regretted that he had been misunderstood in some ways, but was pleased at the generally favorable reception of his paper.

*Stated Meeting, February 7, 1899.*

Dr. Edwin Williams read a paper on "The Diagnostic Significance of Vomiting." The various local and general diseases of which vomiting may be a result or symptom were enumerated, and briefly, the characteristics of the sort of vomiting associated with them described. When no local cause exists uremia should be thought of. The vomiting of intra-cranial tumor or abscess is not accompanied by much nausea, but is of long standing and associated with headache, usually motor and sensory disturbances, and especially choked disc. The vomiting of meningitis is apt to be severe at first and of an expulsive character. In intestinal obstruction the vomited matter is feculent, and vomiting does not relieve the nausea. Vomiting during the latter part of typhoid fever is suggestive of perforation. In ulcer of the stomach the vomit is bright red blood, while in the hematemesis of cardiac and hepatic disease the blood is dark. In cancer of the stomach it resembles coffee grounds. Hematemesis may be due to vicarious menstruation. Vomiting is sometimes a symptom of hysteria, in which case it produces slight depression, being well borne, as are most hysterical manifestations. Vomiting of pregnancy is common, and the whole therapeutic armamentarium has been suggested for its relief. Dr. Williams suggested daily lavage with a warm normal salt solution containing a little cocain and oxalate of cerium. This is to be given early in the morning, and he has seen it do good in some cases. Vomiting in the latter months of pregnancy suggests renal disease. Seasickness is a form of vomiting which is probably not to be controlled. A mixture suggested by Dr. E. M. Holder was mentioned, containing morphin, cocain and belladonna. Vomiting occurs in exophthalmic goiter. It ushers in various febrile diseases, and is often seen in pelvic diseases. Vomiting is the rule after giving an anesthetic. It may be ameliorated by inhaling vinegar, and in a measure avoided by beginning with chloroform and changing to ether when the patient is anesthetized.

Dr. E. C. Ellett spoke of Gunby's suggestion to wash out the stomach after anesthetization to relieve nausea and vomiting.

Dr. Wm. Krauss said that while cocain is a valuable drug to relieve vomiting, if any *habitué* takes more than his usual dose it is very apt to bring on vomiting.

Dr. G. G. Buford said that the causes of vomiting are either central or peripheral. Some causes act in both ways. In exophthalmic goiter the vomiting is due to an irritation transmitted back to the center in the medulla by way of the recurrent laryngeal and pneumogastric nerves. In narcotism from ether or chloroform or an opiate, metabolism is arrested, and the retention of toxins causes vomiting by their action on the center. Washing the stomach removes some of these toxins and dilutes others in the circulation, aiding their elimination by the kidneys.

Dr. J. H. Venn asked for the modern pathology of seasickness, and wanted to know what had become of Dr. Minor's labyrinthine theory.

Dr. E. M. Holder said he had had a good deal of experience with seasickness, but did not know its cause or how to relieve it, except to let it wear off. He spoke of Gerster's suggestion to spray the nares with cocain before anesthesia, to relieve nausea and other unpleasant effects.

Dr. Richmond McKinney has seen one case of seasickness relieved by Skinner's remedy of atropia and strychnia.

The President thought we should utilize vomiting more as a therapeutic measure in children, in whom many illnesses are due to an overloaded stomach.

Dr. Williams disagreed with Dr. Buford as to the arrest of metabolism during anesthesia, and mentioned sweating as one instance where metabolic activity continues. He regards seasickness as an inevitable result of the motion of a vessel in those susceptible to it.

Dr. R. B. Maury made a report of some cases from the "Gynecologic Clinic at the City Hospital in January." He had two cases of cystitis of about one year's duration. One of the women had a child six weeks old, and during the latter part of her pregnancy the cystitis appeared. In both patients internal and local treatment had been tried without effect. Both women were weak and emaciated, and the puerperal one had a temperature of $103\frac{1}{2}°$, and pulse 140, but no disease except that of the bladder, and possibly kidney. Both had pain and painful and frequent urination. In both cystotomy was done, and in both great relief was at once experienced, and in eight or ten days they were practically free from symptoms. The fistula will be left open for a year. We now recognize ureteritis in all these cases, and to illustrate its importance the case of a young married woman was mentioned, who, four years ago, while under a great nervous strain, neglected to urinate for forty-eight hours. When she attempted to empty the bladder she found it very difficult, and from that time her trouble began. She was urinating hourly, and with much pain; there was pain up along the course of the left ureter; the urine contained a little albumen, pus and epithelial cells, but no evidence of a kidney lesion. The cystoscope showed slight cystitis. The right ureter was catheterized and found to contain pus. The urine in the bladder from the left ureter also contained pus. A cystotomy was done ten days ago, and the bladder washed with a solution of borax, boracic acid and salt. She is now completely relieved.

Emmet proposed a simple slit in the bladder, but Dr. Maury finds the following to give better drainage: Pick up a fold of vaginal wall and excise a strip about one-fourth inch wide down to the mucous membrane of the bladder. Slit this on a sound and unite the vaginal and cystic mucous membranes with a continuous catgut suture. This gives an opening which insures good drainage. The medical treatment of cystitis is unsatisfactory. Cystotomy yields good results.

A case of retroversion had been operated on. The patient was a neurasthenic virgin, in whom a pessary gave no relief. When the pessary fails, there are four operative measures at our disposal—ventro-fixation, suspensio-uteri, folding the vaginal ligaments, and Alexander's operation of shortening the round ligaments. That known as suspensio-uteri is usually the best. The uterus is replaced and held by a pessary, the abdomen opened, and two silk sutures passed through the posterior uterine wall near the top of the organ, and then through the peritoneum and subperitoneal fat of the abdominal wall and tied. The peritoneum and fascia are closed by a continuous catgut suture in two layers, and then the skin sutured. Convalescence is usually prompt, and the result good. The adhesions stretch and allow the uterus some mobility, and pregnancy is possible.

The last case was that of a multipara, who conceived in January, 1898. She passed her time in October, and then fetal movement ceased. The patient's health declined, but she was not septic. A

diagnosis of ectopic gestation was made by the interne, Dr. Black. The head was low in the pelvis, the enlarged uterus to the right, and the os high above the pubis. The abdomen was opened, the sac incised and the fetus delivered. The placenta was attached to the right broad ligament, and was desiccated. The sac was tied off and enucleated, and the pelvis packed with gauze to control the bleeding. For two weeks the pulse was 125–135; temperature 102°–103°; the epigastrium flat; no vomiting, and the patient in good spirits and taking nourishment. On the thirteenth day she contracted a bronchitis, and died on the eighteenth day. In this case it would have been better to have opened the sac low down and drained it, and not to have attempted the ideal operation of its removal.

This is the third case of this character Dr. Maury has seen. In the first there was no sac, and the placenta was attached to the intestines. In the second case the pregnancy was intraligamentous.

---

**NOTICE.—This JOURNAL and THE INTERNATIONAL JOURNAL OF SURGERY one year for $1.00 cash, to new subscribers. No out-of-town checks accepted unless 10c. is added for cost of cashing.**

---

DR. J. FARRAR reported some cases to the last session of the British Medical Association to call attention to a rapid method of overcoming a rigid os uteri in labor. It consists in the application of a ten per cent. solution of the hydrochlorate of cocaine on a piece of rag—smearing the os round and round—first on the outside, and then within—finally leaving the rag within the margin of the os. At the end of about four minutes, the os not only loses its rigidity, but is wide open, and as flexible and distensible as a rubber bag. The doctor had had opportunity to test the use of cocaine in only five cases of rigid os in labor before publishing his report; but in each of the five cases the ten per cent. solution of cocaine acted with equal success. This is "a good obstetric wrinkle."

